# Coronavirus disease 2019 is threatening stroke care systems: a real-world study

**DOI:** 10.1186/s12913-021-06297-4

**Published:** 2021-03-31

**Authors:** Jiawei Xin, Xuanyu Huang, Changyun Liu, Yun Huang

**Affiliations:** 1 Department of Neurology, Fujian Medical University Union Hospital, 29 Xinquan Road, Fuzhou, 350001 China; 2 Institute of Neuroscience, Fujian Key Laboratory of Molecular Neurology, Fujian Medical University, 29 Xinquan Road, Fuzhou, 350001 China; 3 Department of Geriatric Medicine, The First Affiliated Hospital of Fujian Medical University, Fuzhou, 350004 China

**Keywords:** COVID-19, Pandemic, Stroke, Stroke care, China, Workflow, Real-world

## Abstract

**Background:**

Since the onset of the coronavirus disease 2019 (COVID-19) pandemic, the stroke care systems have been seriously affected because of social restrictions and other reasons. As the pandemic continues to spread globally, it is of great significance to understand how COVID-19 affects the stroke care systems in mainland China.

**Methods:**

We retrospectively studied the real-world data of one comprehensive stroke center in mainland China from January to February 2020 and compared it with the data collected during the same period in 2019. We analyzed DTN time, onset-to-door time, severity, effects after treatment, the hospital length of stays, costs of hospitalization, etc., and the correlation between medical burden and prognosis of acute ischemic stroke (AIS) patients.

**Results:**

The COVID-19 pandemic was most severe in mainland China in January and February 2020. During the pandemic, there were no differences in pre-hospital or in-hospital workflow metrics (all *p*>0.05), while the degree of neurological deficit on admission and at discharge, the effects after treatment, and the long-term prognosis were all worse (all *p*<0.05). The severity and prognosis of AIS patients were positively correlated with the hospital length of stays and total costs of hospitalization (all *p*<0.05).

**Conclusions:**

COVID-19 pandemic is threatening the stroke care systems. Measures must be taken to minimize the collateral damage caused by COVID-19.

## Background

The coronavirus disease 2019 (COVID-19), which is caused by severe acute respiratory syndrome coronavirus 2 (SARS-CoV-2), has developed into a global pandemic since it first spread in mainland China in late 2019. This pandemic has been causing a global public health event and a global health threat [1], not only due to COVID-19 itself, but also all other diseases which are directly or indirectly affected. As of January 31, 2021, COVID-19 has infected more than 1 billion people worldwide and caused more than 2 million deaths [2].

Stroke patients and stroke care systems are significantly affected during the COVID-19 pandemic. On the one hand, COVID-19 can affect the cardiovascular and cerebrovascular systems [3] by down-regulating the angiotensin-converting enzyme 2 and decreasing the activation of the alternative renin-angiotensin system pathway in the brain [4, 5], which may result in stroke during SARS-CoV-2 infection. On the other hand, some media and doctors noticed a drop in admissions for acute myocardial infarction patients [6, 7] and stroke patients [8], while their morbidities are rarely possible to decline during the pandemic.

There is no doubt that the social restrictions, the government interventions, and many other factors have inevitably affected tens of thousands of stroke patients during the COVID-19 pandemic. To make matters worse, as the pandemic continues, these effects may last for several years or even longer. However, the exact effects on stroke care systems caused by COVID-19 stay unclear, and the conclusions of previous studies remain controversial [8, 9]. In addition, because of the obvious differences in national conditions, medical standards, medical systems, political systems, customs, and cultures among countries, it is necessary to conduct extensive studies on different economies such as China, the United States, the European Union, etc.

The present study aimed to: (a) review the impact of the COVID-19 pandemic on the stroke care systems in mainland China by comparing the real-world data during the pandemic and during the same period in 2019; (b) explore the correlation between changes during the pandemic and clinical outcomes; and (c) better understand the changes of stroke care systems caused by COVID-19 to help make effective and feasible measures and strategies.

## Methods

### Study design and population

Fujian Medical University Union Hospital is a nationally certified acute stroke-ready hospital with one of the biggest regional comprehensive stroke centers in southeastern China. Over 2 thousand acute ischemic stroke (AIS) patients were admitted in 2019. This regional comprehensive stroke center mainly serves the people of Fujian Province (Its permanent population is about 40 million as of the end of 2019 [10]), and it also provides services to several surrounding provinces. We retrospectively analyzed the quality improvement data of AIS patients admitted at the comprehensive stroke center of Fujian Medical University Union Hospital from January to February 2020, which was the most severe period of the COVID-19 pandemic in mainland China. We compared these patients discharged with a principal diagnosis of AIS to patients from the same period in 2019. The study was conducted from data already collected and did not collect any identifiable information on patients. The study was approved by the Ethics Committee of Fujian Medical University Union Hospital. The study data is available from the corresponding author upon reasonable request.

### Quality improvement data

To review and monitor stroke care quality benchmarks, indicators, evidence-based practices, and outcomes, the data of each AIS patient is collected and recorded in detail by the hospital. We queried the demographic data of the patients, whether they accepted thrombolysis and/or thrombectomy and the door-to-needle (DTN) time among thrombolysis, and other clinical characteristics data. Clinical characteristics data include onset-to-door time, the degree of neurological deficit evaluated by National Institutes of Health Stroke Scale (NIHSS), effects after treatment, the hospital length of stays, costs of hospitalization, etc.

### Statistical analysis

Statistical analyses were performed using the Statistical Package for the Social Sciences (SPSS) software version 26.0 (IBM, Armonk, New York). Demographic data, DTN time, onset-to-door time, severity, effects after treatment, the hospital length of stays, costs of hospitalization, etc. were compared between the same period in 2020 and 2019. Continuous variables were compared using the Mann-Whitney U test and the Chi-square test was used for categorical variables. The Spearman correlation analyses were used to detect the associations between clinical characteristics and outcomes. The statistical significance threshold was set to *p* < 0.05. The statistical tests were two-sided.

## Results

**Fig. 1:**
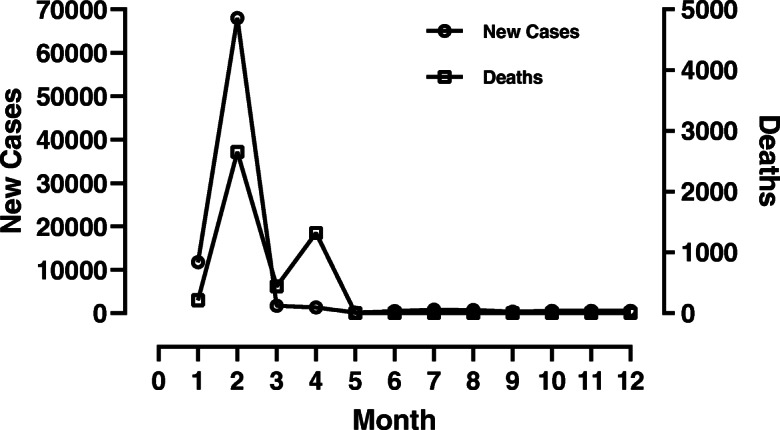
The number of new cases and deaths of COVID-19 per month in mainland China in 2020. On the left axis, the number of new cases of COVID-19 per month in mainland China in 2020 (empty circles) are shown. On the right axis, the number of deaths of COVID-19 per month in mainland China in 2020 (empty Squares) are shown. Data cited from National Health Commission of the People’s Republic of China [11]. COVID-19, the coronavirus disease 2019

The COVID-19 pandemic was most severe in mainland China in January and February 2020 (There were 11,791 new cases and 213 deaths in January, and 68,033 new cases and 2657 deaths in February [11]). New infections and deaths declined quickly after peaking in February (Fig. 1). From January to February 2020, a total of 118 patients were admitted to the Department of Neurology, including 37 AIS patients (31.36%). By comparison, this number was 305 in 2019, including 38 patients diagnosed with AIS (12.46%). In addition, the admission rate of intracerebral haemorrhage (ICH) decreased from 9.51% (29/305) in 2019 to 4.24% (5/118) in 2020. A mandatory COVID-19 testing for all acute stroke patients was implemented in the stroke center since January 2020. All AIS patients were COVID-19 negative on admission and none of them were transferred to a designated COVID-19 hospital for developing relevant symptoms.

**Fig. 2:**
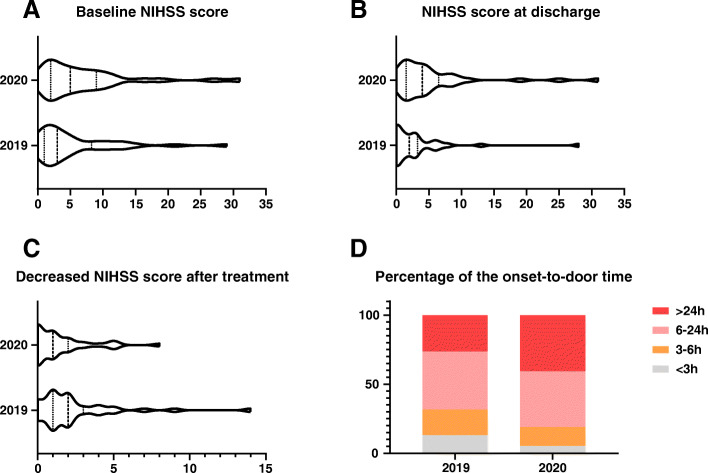
Comparisons of acute ischemic stroke patients between the same period in 2019 and 2020. **a** Baseline NIHSS score. **b** NIHSS score at discharge. **c** Decreased NIHSS score after treatment. **d** Percentage of the onset-to-door time. NIHSS, National Institutes of Health Stroke Scale

During the pandemic, there were no differences in pre-hospital or in-hospital workflow metrics, including the onset-to-door time and the DTN time (all *p*>0.05) (Table 1). However, the baseline NIHSS score of the AIS patients admitted in the pandemic showed a trend high than that in 2019 (Fig. 2a). Moreover, the NIHSS score at discharge was significantly higher and the decreased NIHSS score after treatment was significantly lower during the pandemic than those in 2019 (all *p*<0.05) (Fig. 2b, c & Table 1). Although the number of hospitalized patients decreased by 61.3% compared with 2019, the number of AIS patients was about the same. However, during the pandemic in 2020, only 8.1% of AIS patients arrived at the stroke center within 4.5 h after onset (10.5% in 2019), 18.9% arrived within 6 h (31.6% in 2019), and still 40.5% of the patients did not seek for the stroke care until more than 24 h after onset (only 26.3% in 2019) (Fig. 2d).

**Table 1:** Demographic and clinical characteristics of patients

Variable	2020	2019	P
	(***n*** = 37)	(***n*** = 38)	
**Age (years)**	67 (54, 76)	69 (64, 80)	0.120
**Men, n(%)**	29 (78)	24 (63)	^a^0.206
**Onset-to-door time (hours)**	24 (7, 48)	24 (5, 29.25)	0.227
**Door-to-needle time (minutes)**	53 (24.5, 76.5)	35 (15, 72.5)	0.548
**Baseline NIHSS score**	5 (2, 9)	3 (1, 8.25)	0.235
**NIHSS score at discharge**	4 (1.5, 6.5)	2 (0, 3.25)	0.014^*^
**Decreased NIHSS score after treatment**	1 (0, 2)	2 (1, 3)	0.033^*^
**Hospital length of stays (days)**	12 (9.5, 15.5)	11 (7, 16.5)	0.417
**Total costs of hospitalization (yuan)**	14,423 (10,701, 21,597)	12,084 (9333, 19,348)	0.231

Numbers are n(%) or median (IQR) as appropriate

Continuous variables were compared using the Mann-Whitney U test. ^a^Using chi-square test. *p < 0.05

*NIHSS* National Institutes of Health Stroke Scale, *IQR* interquartile range

**Fig. 3:**
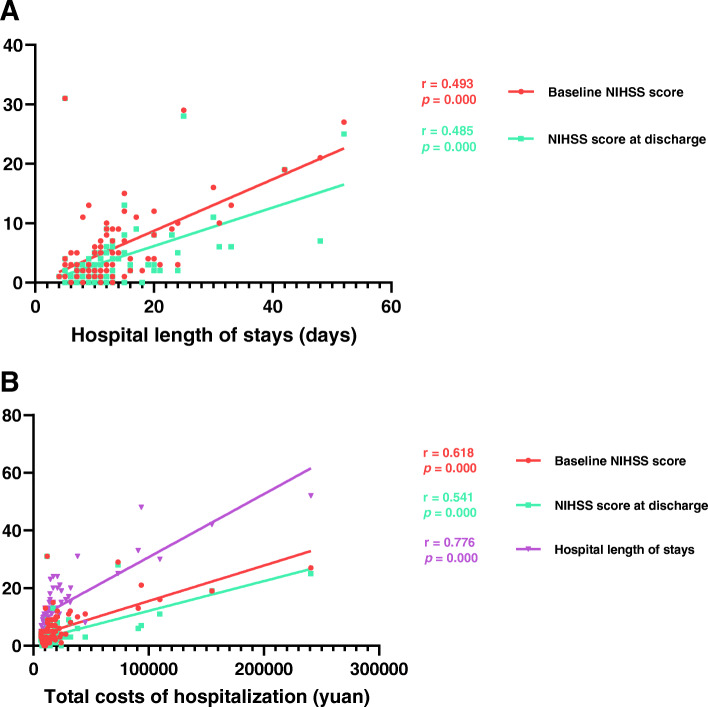
Correlation between medical burden and prognosis of acute ischemic stroke patients. **a** Correlation between the hospital length of stays and the NIHSS score. **b** Correlation between the total costs of hospitalization and the NIHSS score. NIHSS, National Institutes of Health Stroke Scale

Correlation analyses were performed to determine the relationship between medical burden (the hospital length of stays and the total costs of hospitalization) and prognosis of the AIS patients. The hospital length of stays was positively correlated with the baseline NIHSS score (*r* = 0.493, *p* = 0.000) and the NIHSS score at discharge (*r* = 0.485, *p* = 0.000) (Fig. 3a). And the total costs of hospitalization were positively correlated with the baseline NIHSS score (*r* = 0.618, *p* = 0.000), the NIHSS score at discharge (*r* = 0.541, *p* = 0.000), and the hospital length of stays (*r* = 0.776, *p* = 0.000) (Fig. 3b).

## Discussion

### Challenges and changes in stroke care systems

During the pandemic in January and February 2020, extreme measures were taken to prevent the spread of COVID-19. The Chinese government locked down cities and communities, shut down public transportation and most public services, declared temporary suspensions of production and schools, etc., aiming to reduce population movements and gatherings. In the health system, body temperature was strictly monitored. Fever patients were compulsively arranged to an independent procedure called “fever clinic”. After excluding the possibility of COVID-19 infection, the fever patients would then be transferred to the regular medical system. In the meantime, more than 40 thousand doctors and nurses nationwide were sent to Hubei Province, China, to combat COVID-19 [12].

Due to the differences in medical and political systems, the impact of COVID-19 on stroke centers in different countries varied. Different approaches were taken by different economies when facing the pandemic, which depended on their resources and health care system organization. The United States of America is the country with the largest number of COVID-19 infections and the largest number of deaths in the world [2]. Some studies observed a significant decrease in acute stroke presentations across emergency departments at the time of a surge of COVID-19 cases [8]. Another study demonstrated that during the pandemic there was a significant decrease in the use of stroke imaging both in patients with severe strokes and in nonelderly patients who may have been at low risk for COVID-19 complications [13]. In Europe, France, Spain, Italy, and Germany are severely affected by COVID-19, and their numbers of infections rank among the top ten in the world [2]. In Italy and France, because of the reduced capacities of stroke care services caused by COVID-19, stroke care was centralized to a limited number of stroke centers while the remaining stroke units were dedicated to COVID-19 patients, leading to a marked reduction in the number of stoke patients [14]. In Spain, stroke admissions and the number of thrombectomies declined, particularly after the population lockdown [15]. In Germany, decreasing hospital admissions due to ischemic cerebrovascular events were observed as well [16]. It is worth noting that not only the present study in mainland China, but also other studies in the United States and countries of the European Union [8, 15, 16], that the quality of stroke care metrics was not significantly affected, suggesting that global medical workers were still trying their best to provide high-quality stroke care services under the severe burden of the medical system caused by COVID-19.

It seemed the admission of AIS patients did not decrease during the pandemic in mainland China. This may be because: (a) as a regional comprehensive stroke center, the stroke unit remained open during the pandemic; (b) even during the pandemic, patients still tended to visit a comprehensive stroke center rather than the nearest primary stroke center; (c) stroke causes more concern and fear even than COVID-19 due to the serious sequelae and consequences; (d) the public awareness of stroke improved thanks to education endeavors in recent years. In contrary to the admission rate of AIS patients, the admission rate of patients with cerebral hemorrhage dropped by more than a half. A possible explanation of this phenomenon is that ordinary hospitals can be competent for the treatment of patients with cerebral hemorrhage, so during the pandemic patients with cerebral hemorrhage may just choose the nearest hospital. In China, patients can decide by themselves to ignore the system of tiered diagnosis and treatment, and even emergency medical service personnel tend to send patients directly to comprehensive stroke centers for treatment. This also reflects the importance of the construction of stroke centers, the optimization of stroke care systems, and the promotion of the stroke education.

The baseline NIHSS score and the NIHSS score at discharge were both higher than those in 2019, while the decreased NIHSS score after treatment was lower than that over the same period last year, suggesting that the degree of neurological deficit on admission and at discharge, the effects after treatment, and the long-term prognosis were all worse during the pandemic. Correlation analyses showed that both the hospital length of stays and total costs of hospitalization were positively correlated with the baseline NIHSS score and the NIHSS score at discharge. The severity and prognosis of AIS patients were related to the hospital length of stays and total costs of hospitalization. The COVID-19 pandemic directly and indirectly affected the will, time, and capacity of the AIS patients to reach the stroke centers, leading to worse prognosis of AIS patients, which in turn caused negative effects and a higher medical burden.

### Suggestions and strategies for government, medical systems, and patients

As the worst global public health event in recent decades, the COVID-19 pandemic still lingers, and it seems difficult to obtain effective control in the short term. Therefore, it is of great significance to understand how the COVID-19 affects stroke care systems in the real-world and then take effective measures. We recommend the following strategies to be implemented as soon as possible:

For the government: (a) make a prudent decision on the lockdown of cities and communities; (b) keep the public transportation and services in a safe and orderly manner as much as possible, especially the emergency medical system; (c) optimize allocations of medical resources to prevent a collapse of medical system; (d) remind the public even during the pandemic it is highly recommended to visit the nearest stroke center as soon as possible; (e) improve the public education about stroke, encourage primary prevention, advance acute therapy, appreciate secondary prevention and recovery, and reduce regional disparities in the stroke care [17].

For the medical systems: (a) ensure that stroke centers still provide high-quality stroke care during the pandemic; (b) review and optimize the stroke care quality according to the pandemic situation; (c) add the body temperature monitoring, lung CT scan and COVID-19 nucleic acid testing to the standard stroke care system if possible, and ensure that the DTN time of more than 50% of AIS patients treated with IV alteplase is still within 60 min [18]; (d) establish independent passages and isolation wards for stroke patients with fever, a history of epidemics, or suspected COVID-19 infection; (e) provide necessary and sufficient personal protective equipment against COVID-19 for healthcare professionals in stroke centers [19]; (f) prevent healthcare professionals from overwork; (g) use telemedicine in locked-down regions for a timely review of brain imaging in stroke patients, and expedite the decision making process for rapid imaging interpretation in time for IV alteplase [18], etc.; (h) explore the feasibility of high-tech applications for the stroke care during the pandemic, such as unmanned wards, artificial intelligence, big data analysis, virtual reality technology, etc.

For patients: (a) adhere to the primary prevention of stroke, such as the management of hypertension, diabetes, etc.; (b) once early stroke symptoms appear, seek help immediately from the nearest stroke center; (c) provide doctors with detailed medical history, such as epidemiology, the close contact history of patients with COVID-19 infection, etc.

Still, limitations and some methodological issues should be considered. This study was a real-world study which was observational and retrospective. Although the present study has provided some evidence suggesting that COVID-19 posed a threat to stroke care systems, large-sample and long-term follow-up studies are still needed to determine the ultimate impact of COVID-19 on stroke patients and stroke care systems. Besides, Fujian Province (As of January 31, 2021, a total of 541 COVID-19 cases were confirmed [11]) belongs to the moderate epidemic areas (As of January 31, 2021, its confirmed COVID-19 cases are between 100 to 999 [11]). Studies on nationwide cohort are important and persuasive to demonstrate the impacts on the stroke care by anti-contagion policies during the COVID-19 pandemic in mainland China.

## Conclusions

Evidence and data from real-world suggest that the COVID-19 pandemic is threatening stroke care systems. Effective measures must be taken immediately to minimize the collateral damage caused by COVID-19.

## Data Availability

The datasets used and/or analyzed during the current study available from the corresponding author on reasonable request.

## Abbreviations

COVID-19: The coronavirus disease 2019
SARS-CoV-2: Severe acute respiratory syndrome coronavirus 2
AIS: Acute ischemic stroke
DTN time: Door-to-needle time
NIHSS: National Institutes of Health Stroke Scale
